# Not a Steady Drinker

**Published:** 1913-05

**Authors:** 


NOT A STEADY DRINKER.—A doctor's patient in Excelsior Springs the other day was answering the usual list of queries prior to entering upon a course of treatment.

"Are you a steady or a periodical drinker?" asked the physician.

"Periodical," was the reply.

"How long between periods?"

The poor fellow studied a moment, that he might answer correctly, and replied:

"About twenty minutes."

